# Advances in Millimeter-Wave Treatment and Its Biological Effects Development

**DOI:** 10.3390/ijms25168638

**Published:** 2024-08-08

**Authors:** Rui Jing, Zhenqi Jiang, Xiaoying Tang

**Affiliations:** 1 School of Medical Technology, Beijing Institute of Technology, Beijing 100081, China; 3120215946@bit.edu.cn; 2 School of Life Science, Beijing Institute of Technology, Beijing 100081, China

**Keywords:** millimeter-wave treatment, biological effect, non-thermal effect, therapeutic potential, research challenges

## Abstract

This comprehensive review critically examines the current state of research on the biological effects of millimeter-wave (MMW) therapy and its potential implications for disease treatment. By investigating both the thermal and non-thermal impacts of MMWs, we elucidate cellular-level alterations, including changes in ion channels and signaling pathways. Our analysis encompasses MMW’s therapeutic prospects in oncology, such as inducing apoptosis, managing pain, and modulating immunity through cytokine regulation and immune cell activation. By employing a rigorous methodology involving an extensive database search and stringent inclusion criteria, we emphasize the need for standardized protocols to enhance the reliability of future research. Although MMWs exhibit promising therapeutic potential, our findings highlight the urgent need for further elucidation of non-thermal mechanisms and rigorous safety assessments, considering the intricate nature of MMW interactions and inconsistent study outcomes. This review underscores the importance of focused research on the biological mechanisms of MMWs and the identification of optimal frequencies to fully harness their therapeutic capabilities. However, we acknowledge the challenges of variable study quality and the necessity for advanced quality control measures to ensure the reproducibility and comparability of future investigations. In conclusion, while MMW therapy holds promise as a novel therapeutic modality, further research is imperative to unravel its complex biological effects, establish safety profiles, and optimize treatment protocols before widespread clinical application.

## 1. Introduction

Millimeter wave (MMW) is a conventional name; no organization has strictly defined it. MMW is fundamentally recognized as a component of the electromagnetic wave spectrum in the frequency range of 30 GHz~300 GHz and wavelength range of 1~10 mm [1,2]. It was first discovered by the physicist Peter Nikolayevich Lebedev of the former Soviet Union (FSU) in the late 19th century. Research on MMWs stagnated due to the lack of natural sources of MMW radiation in the terrestrial environment. Until the physicist Frohlich proposed the resonance theory, MMW research was not carried out in the medical field. Around the 1970s, with the successful development and mass production of MMW emitters, biological and medical research on MMW progressed along a scientifically rigorous trajectory. Over the past 20 years, research in this field has faced many difficulties and limitations. However, with the help of technological innovations, such as more powerful and efficient MMW emitters and advanced imaging techniques, these advances have overcome the limitations of earlier research. Furthermore, the application of omics technologies (such as genomics, proteomics, and metabolomics) and studies on the combination of millimeter-wave therapy (MMWT) with other treatments have deepened researchers’ understanding of the biological effects of MMW. Scientists from various countries have published more than 100 papers attempting to explain the primary mechanism of the biological effects of MMWs.

As a physical factor, which has already been utilized in anti-inflammatory treatment, immune system stimulation, sedation, and analgesia [3], MMWT has the advantages of low side effects, user-friendly control, and cost-effectiveness compared with traditional therapy. The FSU began researching MMW biological effects as early as the 1960s and determined three optimal frequencies (42.2, 53.6, 61.2 GHz) for MMWT [3]. Consequently, numerous studies have employed these frequencies for experimental purposes. However, it remains unclear why these frequencies were chosen. The non-thermal effects of MMWs are usually studied under a low power density. When the thermal impact occurs, the power density of MMWs is much higher than this, and the research on the mechanism of the thermal effect is relatively mature. The escalating prevalence of MMW technology in communication systems has prompted a nascent yet growing interest in examining its security implications and vulnerabilities. Investigations into the biological effects of MMWs predominantly occur in non-clinical contexts, comprising in vivo and in vitro studies that span healthy and pathologic cell lines and tissue models. MMWs have been shown to elicit a variety of biological effects, including both thermal and non-thermal mechanisms. Thermal effects arise from the absorption of energy by tissues, leading to temperature increases, which can influence cellular processes [4]. Non-thermal effects, on the other hand, involve mechanisms that do not result in significant temperature changes and include alterations in gene expression [5], protein synthesis [6], and cell signaling pathways [7]. These non-thermal effects are of particular interest due to their potential therapeutic applications, such as anti-inflammatory effects, immunomodulation, and analgesic properties. Additionally, MMWs can affect cellular metabolism [8] and DNA repair mechanisms [9] and even have potential anticancer effects. The mechanisms underlying these biological effects remain subjects of ongoing research, but they hold promise for novel therapeutic interventions.

Despite this, other countries have not fully recognized the clinical therapeutic effect of MMW yet. In addition to the quality control problems with a large number of experiments, such as the lack of blinding, dosimetry, temperature, and negative/positive controls, the reasons for this include the lack of in-depth and systematic research on the primary mechanism of the biological effects of millimeter waves. To enhance the credibility and recognition of millimeter-wave therapy, it is necessary to strengthen the exploration and demonstration of the fundamental theories behind its biological effects.

This review aims to comprehensively summarize and critically assess the research on the biological effects of millimeter waves (MMWs) over the past two decades. Given the controversies and uncertainties surrounding the non-thermal biological effects of MMWs, it is essential to synthesize the existing literature and identify gaps in the current knowledge base. The review focuses on synthesizing the existing research, identifying the most promising research directions, and proposing strategies to address the existing controversies and uncertainties in the field. By doing so, we hope to contribute to advancing the field of MMW research and enhancing the credibility and recognition of millimeter-wave therapy. Specifically, this review categorizes and analyzes the research on the biological effects of MMWs, aiming to provide a basis for future studies. Initially, we summarize the recent developments in MMW therapy. Following that, we present and discuss studies on the effects of MMWT on morbid cells or tissues. Finally, the core part of this review classifies and discusses the biological effects of MMWs, with a particular emphasis on seeking direct evidence of the non-thermal biological effects and exploring potential entry points and breakthroughs for subsequent research.

## 2. Methodology

### 2.1. Literature Retrieval Strategy

In order to systematically collect the literature about millimeter-wave therapy and its biological effects, we adopted a comprehensive and meticulous retrieval strategy. First of all, we used computer-aided literature screening tools to conduct an extensive search for many well-known databases worldwide, including but not limited to PubMed, EMBASE, Web of Science, Scopus, and so on. The search keywords were carefully designed to cover all relevant research, and the keyword combination included “Millimeter Wave”, “Biological Effects”, “Treatment”, and related terms. The search time was until 2023 to include the latest research results.

### 2.2. Screening Criteria and Process

The inclusion criteria were strictly limited to original published peer-reviewed research, review articles, or meta-analyses, which should focus on the biological effects and therapeutic applications of millimeter waves and be published in English. We excluded the literature that only involved in vitro studies or had methodological defects in non-animal models and ensured the clinical relevance and practicability of the included studies. The selection process followed the double-blind principle and was carried out by two independent reviewers. Any discrepancies were addressed through discussion or third-party arbitration to ensure the objectivity and scientific rigor of the selected articles.

### 2.3. Quality Assessment and Data Extraction

The selected literature was subjected to a rigorous evaluation process, wherein its methodological quality, reliability of results, and validity of conclusions were analyzed in accordance with the established scoring standards. At the same time, we systematically collated the critical information in the literature, including but not limited to research design, sample characteristics, intervention measures, results indicators, and safety assessment, to ensure the accuracy and integrity of the data.

## 3. Overview of MMWT and Its Biological Effects

MMW represents an extremely high-frequency segment within the electromagnetic wave spectrum, which naturally occurs in the universe. Locating natural sources of MMW isolated on the earth is almost impossible because of the significant atmospheric attenuation experienced during propagation. After the invention of the coherent MMW oscillator in the 1960s, scientists put forward a new train of thought: What impacts does MMW irradiation have on terrestrial organisms? Scientists have started a wide range of research and exploration of the biological effects of MMW.

MMW biological effects have been dominated by studies in the former Soviet Union (FSU) for the past century. In a 1995 study, Lebedeva et al. [4] pointed out that over 3 million people had been treated in more than 1000 MMWT centers in the FSU and that more than 50 diseases could be successfully treated with MMW alone or adjuvant. Three years later, Pakhomov et al. [10] showed that in version 3.01 of the EMF database (1997), 463 publications related to MMW were published by the FSU, approximately two-thirds of the world’s total. In the 2006 research, Marvin C. Ziskin reported that there were three main reasons that other countries have not readily cited the FSU’s remarkable achievements in MMW research: (1) journals lack high-quality peer review; (2) research studies are deficient in terms of quality control measures; and (3) theories lack explanation of systemic effects [3]. Unfortunately, apart from these reasons, the sources of research in the FSU are not easily accessible to Western scientists, making it extremely difficult for other countries to apply and expand in this domain. In addition, due to the limited number of research groups actively investigating MMW, the existing research paradigms might exert disproportionate influence, inadvertently shaping the global research landscape and introducing a particular bias in the collective findings across international studies.

Since the end of the last century, other nations have initiated a comprehensive study and review of the research achievements of the FSU. In 1996, the International Society of Bioelectromagnetism held the first special conference on MMW. In 1997, the conference on electromagnetism and its applications was held. The working conference on infrared laser and MMW was held in the same year. It can be seen that the biological effect of MMW is gradually being paid attention to by the international community. However, from 2006 to the cutoff date of this review in 2024, research questions about MMW’s safety and its biological effects have not been well addressed, and research progress has been slow. Regarding research content, the FSU focused on the biological and medical value of MMW, while the other countries preferred to research the biosafety of MMW. For example, European scientists published a review on MMW and human–body interactions in 2011 [11]. This review focuses on the human body’s biological effects (electromagnetic, thermal, and non-thermal effects, etc.) at the frequency of MMW wireless application (~60 GHz). It analyzes the potential safety hazards of MMW to human beings. 

While the thermal effect of MMW is widely accepted in certain situations, the biological mechanism of its non-thermal effect is still controversial and requires further research to clarify. The non-thermal effect of MMW refers to a series of impacts produced by organisms under the low power density irradiation of MMW. At present, the mainstream theoretical mechanism is divided into two types. Frohlich put forward the coherent oscillation theory [9]. It pointed out that the biological tissue components in the human body, such as the cell membrane, protein, and DNA, which have an inherent oscillation frequency, will resonate when irradiated by MMW and thus lead to a series of non-thermal biological effects. This is also a theory that most scholars tend to accept. The Acoustics hypothesis posits that the cell membrane will oscillate in a manner analogous to sound waves when exposed to millimeter wave radiation. This phenomenon is postulated to result in the rapid reorientation of water molecules inside and outside the cell, thereby accelerating the metabolic processes of the cell [8,11,12,13]. The resonance of water molecules is the subject of both theories. Other theories, such as the acupoint effect of MMW, cannot be fully verified due to a few related studies [14,15]. Based on the above theories, the non-thermal biological effect of MMW may also be power-dependent. According to the study of using tumor polypeptide antigen (TPA) to inhibit the gap junction intercellular communication of HaCaT keratinocytes in 2003 [16], MMW irradiation on cells completely reversed the inhibition of TPA at a power density of 3.5 mW/cm^2^ but partially reversed it at a power density of 1.0 mW/cm^2^. It can be concluded that the non-thermal biological effect of MMW may be linked to its power density. Moreover, theoretically, there may be a threshold for irreversibility.

## 4. Possibility of MMWT in Diseases

The treatment of diseases with physical factors has always been a hot research topic in the field of biomedicine. MMWT has been the subject of extensive research in the FSU since the 1960s due to the benefits it offers in terms of low side effects and non-invasiveness. Many MMW research institutes and treatment centers have been opened. However, some of its research in this field is not publicly available, and the results of the available public research have shown that research on the treatment of malignant diseases has not reached the clinical stage. Up to now, the leading theory about the therapeutic mechanism of MMW is still its thermal effect or resonance effect on tissues or cells, and there is still a lack of clear understanding of the interaction mechanism between MMW and organisms in various countries. If the mechanism of MMW action is sufficiently studied, MMWT will emerge with preferable maneuverability, targeting, and universality in clinical medicine. The objective of this section is to present a critical analysis of the existing research on the non-thermal effects of MMW on the treatment of various diseases, including tumors and cancer. This section will review research on cancer and tumors as a focus.

### 4.1. In Vivo and Vitro Studies of Cancer and Malignancy

MMWT research for cancer and malignant tumors primarily takes place in the experimental phase, emphasizing isolated biological systems, such as molecular, cellular, and animal models. Due to the shallow penetration depth of MMW, it tends to act directly on the epidermis and dermis layers. Thus, there are many studies on treating superficial skin cancer with MMW. As early as 2004, Radzievsky et al. [17] indicated that MMWT could inhibit melanoma growth, and naloxone pretreatment could completely offset the growth inhibition. Naloxone is an opioid antagonist that competes for opioid receptors. This indicates that MMWT has anticancer therapeutic potential, and endogenous opioids are involved in the MMW-mediated treatment process. Irradiation with broadband millimeter waves spanning 53.57 to 78.33 GHz notably inhibited the proliferation of human melanoma cells. In contrast, exposure to single frequencies at 51.05 GHz and 65.00 GHz did not significantly alter cell growth [13]. Subsequent studies have obtained similar results: low-power MMW irradiation with a single frequency of 42.2 GHz and 53.57 GHz will not change the doubling time and cell cycle of RPMI7932 human skin melanoma cells [18]. In addition, the human melanoma cells irradiated by broadband MMW will undergo some morphological changes related to osmotic transmembrane balance and the water molecules in the cells. The latest research pointed out that MMW can inhibit the vitality of A375 cells by activating Casp-3 and Caspase-8 (Casp-8), thereby promoting cell apoptosis [19]. This study explains the specific mechanism of MMWT for superficial cancer.

In addition to MMWT irradiation studies targeting cancer cells in the superficial layer of the skin, there are also studies targeting other distant cancer cells. In an investigation of MMW-irradiated human breast cancer MCF-7 cells, the authors observed a suite of ultrastructural alterations, including compromised nuclear and plasma membrane integrity, mitochondrial swelling, and dilation of the rough endoplasmic reticulum, indicative of cellular dysfunction and potential apoptotic pathways [20]. MMW irradiation of H1299 human lung cancer cells with W-band (75–105 GHz) would increase its aging and death rates within 7–14 days after exposure [21]. MMW irradiation has also been proven to promote the apoptosis of SW1353 human chondrosarcoma cells [22]. It seems that the occurrence of these distant effects should be related to cellular signaling pathways, but few studies have involved cellular pathways in organisms. Data show that MMW can indirectly inhibit distal cancer cells and tumors by changing the cytokine content in cell signaling pathways. For example, MMW could (1) enhance the activity of T cells, (2) enhance the cytotoxicity of macrophages, (3) inhibit the drug resistance of tumors, and (4) inhibit tumor metastasis by enhancing the activity of natural killer cells (NK cells) [23,24,25,26]. Table 1 summarizes the parameters and conclusions of the research mentioned above.

In fact, several investigations of MMW irradiation’s potential to suppress cancer have produced conflicting or unfavorable results. In 2002, Logani et al. [30] indicated that MMW irradiation could not alleviate the immune system inhibition induced by cyclophosphamide (CPA). The results make it clear that MMW exposure cannot enhance the activity of T cells, which is contrary to the results of many subsequent studies [24,25,27]. The majority of studies conducted in recent years have revealed that MMW exerts biological effects on the proximal or distal end of cancer cells or tumors. This challenges the earlier hypothesis that MMW would not be an effective treatment for cancer or tumors. Despite the paucity of research examining cell pathways in organisms, the precise methodology may entail the investigation of cell signaling pathways.

### 4.2. In Vivo and Vitro Studies of Other Diseases

Currently, MMW therapy has potential applications in the treatment or symptom relief of a wide range of diseases, such as inflammation, arthritis, pain management, certain blood disorders, and novel coronavirus infections. Evidence shows that histamine, arachidonic acid, and other polyunsaturated fatty acids are involved in MMW anti-inflammatory treatment [31,32]. Histamine has a significant effect on the regulation of inflammation, and polyunsaturated fatty acids like arachidonic acid are lipid messengers of inflammation. MMW irradiation changes the content and proportion of these two substances, leading to a shift in signal conversion and gene expression between cells, thus realizing the anti-inflammatory mechanism of irradiation. Since MMW irradiation affects an organism’s nervous system (as will be discussed in more detail in the following sections), MMW is typically used to relieve pain and itching. Studies manifested that MMW exposure alleviated the scratching behavior of mice, which was caused by itch-causing compounds [33]. This phenomenon was attributed to the fact that MMW can trigger the release of opioids in organisms. Two years later, the same research team conducted a cold water flick test on mice and concluded that MMW had an analgesic effect on organisms [34]. The same conclusion was also put forward in a study in 2008. In this study, the mice were exposed to three specific treatment frequencies (42.25, 53.57, and 61.22 GHz) for the cold water tail-flick test and steel wire surface test, and it was concluded that (1) endogenous opioids were involved in the process of MMWT-induced hypoalgesia; and (2) the effect of MMWT-induced hypoalgesia was dependent on MMW frequency [35]. In a study of MMW irradiation of rabbit knee cartilage, Xia et al. [36] indicated that MMW treatment reduced chondrocyte apoptosis, which correlated with decreased expression of caspase-3 and MMP-13, demonstrating the therapeutic benefit of MMW for arthritis. Other studies have discussed the mechanism of the MMW analgesic effect; some of them believed that the peripheral nervous system is involved in this effect (depending on the exposed site) [37], while some considered that both peripheral and central mechanisms are involved in the analgesic effect [38]. It has also been stated explicitly that MMW may inhibit neuronal excitability in the trigeminal nerve in the spinal cord, resulting in analgesia. Still, the mechanism of this effect needs further experimental evidence [39]. All of these studies have shown that MMW can have distal effects on organisms by affecting the release of neurotransmitters and opioids to achieve neural inhibition. However, a few clinical studies have demonstrated that MMW irradiation has a pain-reducing effect by triggering changes in diastolic blood pressure, leading to activation of blood pressure-regulating regions in the brainstem [40]. 

Millimeter waves have also been found to have biological effects on certain hematological disorders. For example, MMW irradiation on human erythromyeloid leukemia cell line K562 will lead to (1) a decrease in cell number, (2) a change in nuclear-cytoplasmic structure, (3) a change in cell ultrastructure, and (4) an increase in cell glucose metabolism [8]. The main target of MMW irradiation is water in the biological system, and the water molecules absorbing MMW energy will lead to a balance deviation between free water and bound water, thereby affecting the system’s chemical control and cellular processes. 

Moreover, Chinese scientists have applied MMW to the clinical treatment of a new coronavirus (COVID-19) infection. With MMW acupoint treatment, the patient’s clinical symptoms visibly improved [14]. The data showed that within 14 days after receiving MME therapy, the subject patient’s clinical symptoms were relieved, including increased blood oxygen saturation, lung CT change, decreased cough times and body temperature, a slow decrease in lactate dehydrogenase, and the nucleic acid negative rate reached 91.5% after 14 days. The data profile shows that MMW enhances the patient’s immune function and proves that MMW radiation treatment in lung and acupoint combined with traditional Chinese and Western medicine has specific clinical value in treating COVID-19. 

In conclusion, MMW can alter the release of neurotransmitters and opioids to achieve antipruritic or analgesic effects and can be used as adjuvant therapy for other diseases by enhancing the immune function of organisms. In addition, MMW radiation can affect cell membrane structure, cell morphology, cell cycle, and cell signal transduction of organisms, thus affecting how nerve cells and immune cells operate. However, the specific mechanism of these changes needs further investigation.

## 5. Study on the Mechanism of MMW Thermal Effect

The wavelength of MMW is between the overlapping range of microwave and far-infrared waves. Thus, it has the characteristics of both. The principle of thermal effect caused by exposure to MMW is similar to that of microwaves; that is, disordered polar molecules turn along the direction of the external electric field so that intermolecular collisions generate kinetic energy, which leads to thermal effects. The content of water, a polar molecule, accounts for more than 50% of human skin and 60–70% of human body weight. Therefore, it is evident that MMW exposure is more likely to give rise to thermal effects in organisms. MMW exposure with high power density and frequency is more likely to produce thermal effects on organisms than non-thermal effects. Scientists have extensively researched the thermal effect mechanism of MMW, including in vivo and in vitro experiments on cells and animals, including the influence on molecules, cells, tissues, organs, systems, and organisms.

The analysis of the molecular level has yielded certain results, according to researchers. Caspase-3 (Casp-3) is an essential shearing enzyme in the process of apoptosis. However, heat shock protein HSP27 can inhibit the activity of Casp-3 to improve cellular thermotolerance. Studies have shown that the thermal effects of (58.4 GHz) continuous or pulse amplitude-modulated MMW irradiation on melanoma cells in vitro both trigger Casp-3 activation and HSP27 phosphorylation. In contrast, the pulse wave exposure makes the changes in these two proteins more apparent [28]. However, in vivo experiments on mouse skin melanoma showed melanoma cells are more power-sensitive than keratinocytes during MMW hyperthermia [41]. That means the selective death of melanoma cells can be induced under specific output power without harming healthy cells [42]. A study from 1981 showed that when the temperature of monolayer cell culture reached or exceeded 44.5 °C, MMW irradiation would irreversibly destroy the cell ultra-microstructure [43]. However, changing the MMW power density or frequency below this threshold will not affect the cell structure. This indicates a temperature threshold limit for the MMW thermal effect. In addition, thermal effects also have a series of impacts on the nervous system. Alekseev et al. [44], in their 1995 study, claimed that MMW in the frequency range of 54–76 GHz resulted in a slight decrease in lipid bilayer capacitance and a small increase in ionic current. They indicated in the subsequent studies that the temperature increase caused by MMW irradiation would inhibit the activity of neurons and change the amplitude and dynamics of Lymnaea neurons’ discharge current [45,46]. However, heating by a high-temperature heat source will make neuronal activity more frequent, revealing that the thermal effect of MMW is different from that of heat conduction on organisms [47]. In summary, MMW can directly or indirectly affect the thermal effect of protein or cell activity in vivo, resulting in proximal or distal effects. At the same time, the thermal effect can target the diseased cells according to the characteristics of different cells and tissues with varying levels of tolerance to MMW. The parameters of the above research are summarized in Table 2 below, but unfortunately, there are no regular patterns to draw from it.

From the perspective of public safety, the thermal effects of MMW on healthy cells or tissue, and indeed organs, have been the subject of extensive research. Several studies considered that high-power density MMW irradiation would lead to chronic or acute damage to organisms [48]. They concluded that while, in general, the depth of penetration of MMW into biological skin is shallow (≤0.4 CM) [49,50,51], the local MMW irradiation with enough power density will have an impact on the thermosensitive structure in the skin and either its underlying tissues [52], thus leading to circulatory failure and even the death of organism [53]. Long-term exposure to MMW has been proven to cause severe hypotension and even death, which may be caused by high levels of serum glucose and varying degrees of stress, creatinine, and uric acid in organisms irradiated by MMW [54,55]. As the tolerance of eye cells to MMW is lower than that of normal cells, some groups have carried out MMW safety research on animal eyes. In 2009, Kojima et al. [56] pointed out that a horn antenna and two lens antennas (6 mm and 9 mm in diameter), which could emit 60 GHz MMW, would bring different kinds and levels of thermal damage to its cornea. In 2018, they developed a model of ocular injury caused by continuous MMW at 40, 75, and 95 GHz and evaluated the process of ocular injury caused by MMW at different frequencies [57]. The study results showed that various degrees of corneal epithelial damage, corneal edema, and corneal clouding were induced due to the absorption of heat generated by MMW energy by the cornea. Meanwhile, many studies have obtained nearly the same research results, including numerical simulation analysis of the antecedent experimental data [58,59]. In a 60 GHz MMW irradiation study of Xenopus laevis oocytes, the investigators observed changes in the kinetics and activity levels of voltage-gated potassium-sodium channels, as well as sodium-potassium pumps, which accelerate the generation rate of action potential in oocytes, supporting a role based on a thermodynamic mechanism [60]. Nonetheless, some studies have certified that MMW has few effects on the thermal effects of organisms. For instance, in the latest research in 2020, the high-frequency short-pulse MMW was applied to irradiate healthy mice. Still, no physical, physiological, or pathological parameters or behavior abnormalities were observed [61]. There has always been a debate about whether the thermal effects of MMW have a pernicious influence on healthy cells or tissues. Unfortunately, these studies only describe the phenomena, not the entire mechanism’s explanation. This experimental result, which lacks gradient and parameterization, cannot provide sufficient theoretical support for completely excluding the influence of thermal effect in the study of MMW non-thermal effect.

**Table 2:** Examples of MMW thermal effects research.

Cell/Tissue/Animal	Frequency	Power Density or SAR	Exposure Duration/Target Temperature	Research Group	Publication Year
Artificial lipid bilayer membrane	54–74 GHz	2000 W/kg	5–25 min CW and Pulse modulation irradiation	Alekseev et al. [44]	1995
BP-4 pacemaker neuron of the pond snail Lymnaea stagnalis	75 GHz	-	12–22 min	Alekseev et al. [45]	1997
Human forearm and middle finger skin	42.25 GHz	55 and 208 mW/cm^2^	up to 10, 20 min	Alekseev et al. [52]	2005
Lymnaea neurons	60.22–62.22 GHz and 75 GHz	-	5, 10, 20 min	Alekseev et al. [46]	1999
Sprague-Dawley rats	35 GHz	75 mW/cm^2^	under a lethal temperature	Frei et al. [53]	1995
Healthy C57BL/6 mouse skin	101 GHz PW	0.5–1.5 kW	pulse duration 5–10 μs, 20–50 pulses	Furman et al. [61]	2020
Differentiating neuron-like cells	60.4 GHz	10 mW/cm^2^	24 h	Haas et al. [62]	2016
Neuron-like cells PC12 cells	60.4 GHz	10 mW/cm^2^	24 h	Haas et al. [63]	2016
NGF-treated PC12 cells	60.4 GHz	5 mW/cm^2^	24 h	Haas et al. [64]	2017
Primary culture of human keratinocytes	60 GHz	20 mW/cm^2^	3 h	Habauzit et al. [12]	2014
Rats skin	35 GHz	75 mW/cm^2^	0, 19, 38 min	Jauchem et al. [54]	2016
Dutch rabbit eyes	60 GHz	475 or 1898 mW/cm^2^	6, 30 min	Kojima et al. [56]	2009
Rabbit closed eyelid	40, 60, 75, 90 and 162 GHz	≈233 mW/cm^2^	6 min	Kojima et al. [58]	2022
Rabbit eyes	40, 75 and 90 GHz	10–600 mW/cm^2^	6 min	Kojima et al. [57]	2019
Skin cancer in DMBA-initiated SENCAR mice	94 GHz	1 or 333 mW/cm^2^	10 s/time, 2 times/week, 12 weeks	Mason et al. [65]	2001
Rats skin	35 GHz	75 mW/cm^2^	3–6 h or 24	Millenbaugh et al. [48]	2008
Melanoma cells	58.4 GHz CW or PW	3.7 W	49.2 °C	Orlacchio et al. [28]	2019
Keratinocyte and melanocyte cell lines	60.4 GHz	1–20 mW/cm^2^	20 min, 1 h, 6 h, 16 h, 24 h	Quément et al. [66]	2014
Leech interneuron	60 GHz	around 100 mW	≤40 °C	Romanenko et al. [67]	2019
Midbody ganglion nerve cells	60 GHz	1, 2, 4 mW/cm^2^	1 min	Romanenko et al. [47]	2014
Xenopus spinal cord neurons	94 GHz	31 mW/cm^2^	0.2–1 s	Samsonov et al. [68]	2013
Xenopus laevis oocytes	60 GHz	1–600 mW/cm^2^	1.66 min	Shapiro et al. [60]	2013
BHK-21/C13 cells	41.8, 74.0 GHz	320, 450 mW/cm^2^	42.0–44.5 °C	Stensaas et al. [43]	1981
Macrophage cells	35 GHz	75 mW/cm^2^	41–42 °C	Sypniewska et al. [23]	2010
B16F10 murine melanoma cells	42.25 GHz	0.74–1.48 mW/cm^2^	30 min	Szabo et al. [41]	2004
Human epidermal keratinocytes-HaCaT	42.25 GHz (with high IPD), 61.2 GHz (with low IPD)	29 mW/cm^2^ (low IPD), 1.67 W/cm^2^ (high IPD)	30, 60 min	Szabo et al. [69]	2001
Neurons derived from mouse embryonic stem cells	94 GHz	18.6 kW/m^2^	60 min	Titushkin et al. [70]	2009
Human venous blood	32.9–39.6 GHz	10 mW/cm^2^	15 min	Vlasova et al. [71]	2018
Rats skin	35 GHz	0.5–7.5 W/cm^2^	0.5 min	Xie et al. [55]	2011

## 6. Study of Mechanism of MMW Non-Thermal Effect

Whether to treat diseases or biosafety research, the study of MMW biological effects is an issue in the biomedical field. Researchers began studying the non-thermal effects of MMW a long time ago [72]. Various studies have shown that MMW irradiation can change the process of the cell membrane, cell morphology, cell cycle, and cell signal transduction. With the popularization and application of MMW in various fields, whether MMW can induce irreversible injuries to healthy organisms is also the research content that scientists pay attention to. This section will review MMW’s biological effects on the cell membrane, cell morphology, cell cycle, and cell pathway. 

### 6.1. Cell Membrane Structure

As key transmembrane protein components, ion channels in cell membranes are responsible for regulating the flow of ions in and out of the cell, essential for maintaining cell membrane potential and ensuring cellular homeostasis. Given that they are widely involved in fundamental life activities, such as nerve conduction, muscle contraction, and heartbeat, and are closely related to the development of numerous diseases, exploring how MMW affects the function of cell membrane ion channels has become one of the central pathways to unravel the mechanism of their biological effects.

Some early studies showed that the thermal effect dominated the change from MMW on potassium and calcium currents of snail neurons [46]. Millimeter-wave irradiation elevated both ionic currents (peak amplitude, activation, and inactivation rates) in Lymnaea neurons, and these changes disappeared with the addition of ethanol, which attenuates the thermal effect, suggesting that the changes were mainly caused by heating. 

**Figure 1:**
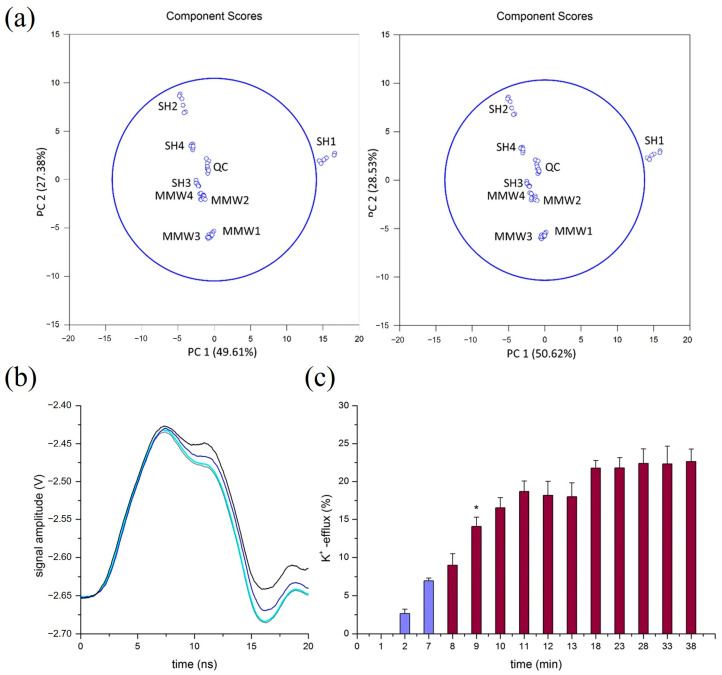
(**a**) PCA plots obtained from intracellular metabolomics analyses of these features showing fold change ≥ 2 and *p*-value < 0.05 after R-XCMS data processing of UHPLC-HRMS sequences recorded in positive ion mode (left) and negative ion mode (right). Copyright 2019, Springer Nature [73]. (**b**) Temporal sequences of electrical signals as affected by valinomycin under EMF, with time-phase coloring demonstrating the effect of the treatment. (valinomycin added at time 0 (grey), at time 7 min (cyan), at time 28 min (blue) and after addition of Triton-X100 at the end of experiment (black).) Copyright 2018, Springer Nature [76]. (**c**) Normalized mean ± SD of potassium ion efflux rate (n = 6), EMF exposure started at 7 min (violet-colored bars) after valinomycin addition and lasted for 30 min (wine-colored bars); *t*-test showed a significant difference between 8 and 9 min (* *p* = 0.028), [K2SO4] concentration was 0.21 mM ± 0.06 based on the calibration curve. Copyright 2018, Springer Nature [76].

The subsequent studies mainly considered that the non-thermal biological effect of MMW changed the structure and function of the cell membrane. In the metabolomics study of human HaCaT keratinocytes irradiated with 60 GHz MMW, MMW radiation dramatically changed the extracellular metabolite sequence [73] (Figure 1a). In particular, the magnetic field components in MMW may affect biological systems through mechanisms similar to weak magnetic field effects. Among them, the kinetic regulation of radical pair complexes is a possible mechanism for the biological effects of weak magnetic fields [74]. Magnetic fields can affect cellular metabolic processes by modulating the spin state of radical pairs and, thus, the probability of radical complexation [75]. However, the upstream pan-transcriptomics in this cell system does not show any change, so it is reasonable to believe that this sequence disorder is not the change in gene expression but the change in cell membrane permeability. 

The cell membrane permeability in eukaryotes is mainly determined by three transmembrane transport modes: passive, active, and vesicle. In comparison, recent research primarily focuses on the latter two modes. A study in 2018 explored the mechanisms by which low-intensity, high-frequency electromagnetic fields (EHF-EMF) affect neuronal activity using an artificial axon model and found that exposure to EHF-EMF (53.37 GHz, 39 mW) increases transmembrane potassium efflux and promotes electrical signal propagation [76] (Figure 1b,c). Li et al. [77] pointed out that MMW regulates the ion channels of chondrocytes bidirectionally by up-regulating the mRNA and protein of several potassium channel-related factors (Kcne1, Kcnj3, and Kcnq2). Meanwhile, MMW irradiation (94 GHz and different power densities) will increase the activity and peak frequency of intracellular Ca^2+^, and the change in calcium spiking depends on the power density of MMW [70,78]. Moreover, MMW irradiation restricted calpain activation by inhibiting intracellular calcium ions’ concentration, thereby regulating the apoptosis of healthy cells [79]. Weak magnetic fields can affect intracellular calcium ion concentration by regulating the opening of voltage-gated calcium channels (VGCCs), which, in turn, affects downstream signaling and gene expression processes [5]. Among them, the STIM1 protein on the endoplasmic reticulum may act as a cellular magnetic field receptor to regulate intracellular calcium ion concentration and oxidative stress, among other processes [80]. Last, MMW exposure would change the Hill coefficient and apparent affinity of calcium channels to calcium ions [81]. This result is also attributed to the destruction of the synergy of binding sites in a single calcium channel. In conclusion, although existing studies have revealed that MMW affects cellular functions through the modulation of ion channels, suggesting a possible specific modulation of cell membrane calcium channels by magnetic field components in electromagnetic waves, there is still a lack of dedicated studies on the direct effects of millimeter-wave-specific magnetic field components and their mechanisms.

Some studies indirectly confirmed the effect of MMW on cell membranes. For instance, MMW irradiation will externalize phosphatidylserine molecules (PSs), resulting in restrictive coagulation [1]. PS externalization refers to the turnover/redistribution of PS from the inner leaf layer to the outer leaf layer of the cell membrane, which plays a vital role in the early stage of thrombosis and apoptosis. In addition, the biofilm has a closed bilayer vesicle structure. MMW irradiation could lead to the reorientation of macrovesicle movement, and the movement direction might be related to the exposure time [82]. All these studies have indirectly proved that MMW can produce a series of effects on the structure and liquidity of biofilm.

### 6.2. Cell Morphology and Cell Cycle

**Figure 2:**
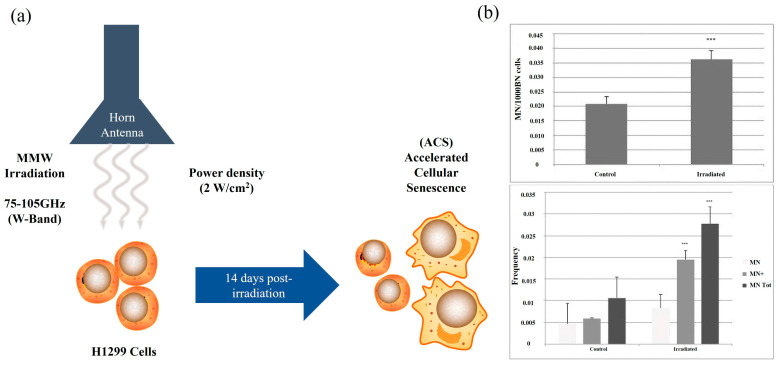
(**a**) Program diagram of H1299 human lung cancer cells irradiated by W-band 75–105 GHz millimeter-waves. (**b**) The micronucleus increased significantly after MMW irradiation (figure above). CREST staining showed that compared with the control group, the frequencies of CREST positive (MN+) and negative (MN−) micronucleus in irradiated samples increased significantly (figure below). *** *X*^2^ test *p* < 0.001. Copyright 2015, Elsevier Ltd. [84].

It has been proved that both tumor and healthy cells exposed to MMW will bring about morphological changes. Morphological changes occurred in human melanoma cells under broadband irradiation (53.57–78.33 GHz), manifested as increased overall cell size and decreased karyocytoplasmic ratio [13]. W-band MMW (75–105 GHz) exposure showed specificity and irreversibility for the morphological changes of cells and nuclei of H1299 human lung cancer cells and was accompanied by significant increases in mortality and senescence rates of MMW within 7–14 days after exposure [21,27] (Figure 2a). In addition to observations of morphological changes in cancer cells exposed to MMW, there has also been some progress in cellular morphological studies of healthy cells. MMW exposure decreases nuclear charge in human oral epithelial cells and aggravates chromatin condensation in the nuclei [83]. Similar results were observed in chondrocytes as well as in human fibroblasts. Chondrocytes exposed to MMW exhibit morphological changes such as increased vesicles, the disappearance of the nuclear membrane, and chromosome aggregation, while human fibroblasts show significantly increased reversible micronuclei [77,84] (Figure 2b). Reversible or irreversible specific morphological changes in cells can be caused by MMW, which can inhibit the growth of diseased cells or change the function of healthy cells.

The influence of MMW on the cell cycle was studied by the same research team, which may lead to distortion in this study area. Li et al. [85] proved that MMW could induce the expression of CDK2 and cyclin A, thus accelerating the cell to enter the S phase and G2/M phase transition. The data showed that after irradiation, the percentage of chondrocytes in the G0/G1 phase decreased, while the number of chondrocytes in the S phase increased significantly. This indicates that MMW promoted chondrocyte proliferation by accelerating the cell cycle process [86]. MMW accelerated the cell cycle by affecting potassium channels [77]. They attributed this change to the cell membrane depolarization caused by the change of extracellular ion concentration, that is, the change in membrane potential, which will regulate the transformation and progress of cells in the G1/S phase and G2/M phase. In conclusion, MMW could induce healthy cell cycle progression and demonstrate the possibility of MMW therapy in promoting cell proliferation.

### 6.3. Cell Signal Pathway

Signal pathway refers to the pathway through which extracellular signal molecules cross the cell membrane and exert biological effects in the cell. Therefore, the study of cell signaling pathways after MMW irradiation can be understood as an essential study of the biological impact of MMW. Although some research results have been accumulated in this field, the effect of MMW on cell signaling pathways cannot be fully elucidated due to the large number of cell signaling pathways and their interactions.

**Figure 3:**
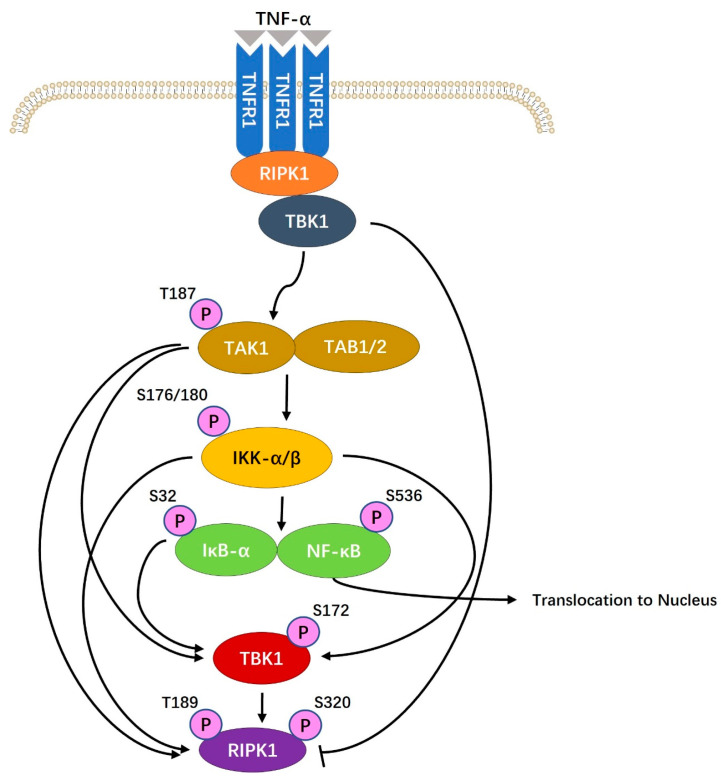
Cell signaling pathway involving TNF-α in activating NF-κB. In the block diagram on the right, TNF-α can activate NF-κB signal and lead to chondrocyte apoptosis. However, MMW irradiation could down-regulate RIP, TAK1, IKK-β, and NF-κB (p65) and up-regulate IκB-α, thereby inhibiting NF-κB signaling and reducing chondrocyte apoptosis.

Li et al. [7] suggested that MMW irradiation could inhibit the NF-κB signaling pathway activated by TNF-α, thus reducing chondrocyte apoptosis. Studies confirmed that the content of signal molecules such as RIP, TAK1, IKK-β, and NF-κB (p65) in the pathway was reduced due to MMW irradiation, thus inhibiting the decay of chondrocytes (Figure 3). In the same year, they suggested that MMW irradiation could also inhibit the apoptosis of chondrocytes induced by SNP [85]. MMW reduces the number of chondrocytes in the process of apoptosis by regulating the activation and expression of p38, p53, and Casp-3 signaling molecules. Mitochondrial-dependent apoptosis is one of the more common apoptotic pathways in vertebrate cells. MMW exposure limits mitochondrial membrane potential collapse, PS externalization, and the activities of Casp-9 and Casp-3 in the pathway to achieve the effect of inhibiting mitochondrial-dependent cell apoptosis pathways in chondrocytes [87]. However, studies also pointed out that MMW could lead to the loss of mitochondrial membrane potential and up-regulate Bax, Casp-9, and Casp-3 cytokines without changing Bcl-2 levels [22]. The data support that MMW pulse irradiation could make the mitochondrial electron transport chain (ETC) of animal cells abnormal, manifested as an increase in the generation rate of superoxide radicals, cell hypoxia, and aggravating metabolic changes [88]. In addition, chemical agonists combined with MMW irradiation could activate the TRPV1 pathway for a long time, leading to apoptosis and affecting leeches’ nociceptive lateral sensory neurons by affecting the sensitization of the TRPV1 pathway [67]. It has been proposed by Szabo et al. [69] that exposure to MMW increases the level of IL-1β, a messenger molecule in HaCaT keratinocytes, thereby inducing distal effects. Studies on such cells also showed that MMW could reverse the inhibition of TPA-induced gap junction intercellular communication (GJIC) [16]. A 2016 study found that six genes associated with intercellular signaling (SOCS3 and SPRY2), cellular stress pathways (TRIB1, CSRNP1, and PPP1R15A), and regulation of cellular value-addition and differentiation (FAM46A) were sensitive to MMW irradiation [89]. The effects of MMW on cellular signaling pathways have also been explored indirectly. In [90], non-thermal MMW-treated saline was used to regulate the hydration status of myocardial tissue through the intracellular cAMP/cGMP signaling pathway. The studies above have confirmed that MMW irradiation could exert specific effects on cellular pathways, such as immune cell signaling pathways, programmed cell death pathways, and neural signaling pathways. However, the research still has many limitations and shortcomings that must be verified in future studies.

## 7. Research on the Effect of MMW on the Nervous System and Immune System

### 7.1. Nervous System

**Figure 4:**
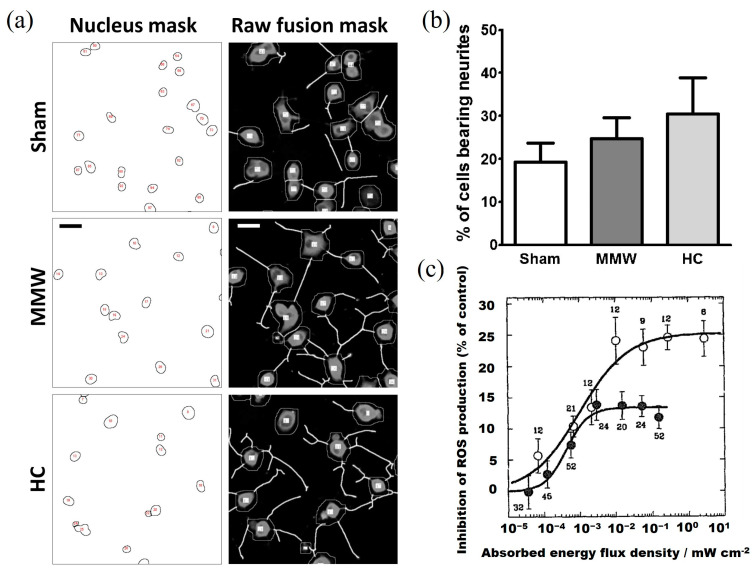
(**a**) The effect of MMW exposure on the differentiation score of PC12 treated by NGF, in which the differentiation score induced by MMW exposure increased slightly, which was related to the thermal effect. Sham (control group), MMW (millimeter-wave irradiation group), and HC (thermal control group). Scale bar = 10 µm. Copyright 2016, Elsevier Ltd. [63]. (**b**) Data in figure (**a**). N = 7, mean of means (error bars = SEM). Copyright 2016, Elsevier Ltd. [63]. (**c**) The suppression of ROS depends on the energy flux density absorbed in the near-field region (hollow circle) and the far-field region (solid circle) of the radiator. Copyright 1997, Elsevier Ltd. [98].

The outermost layer of the organism’s skin will absorb almost all of the MMW exposure, while the dermis and epidermis of the skin surface are highly dominated by the nervous system [91]. An initial investigation demonstrated that MMW at 41.34 GHz frequency can markedly influence the refractory period characteristics of nerves. This effect is contingent upon the frequency of the radiation rather than the intensity of the radiation itself [92]. Subsequent studies reported the thermal impact of 94 GHz MMW on neuronal microtubules [68]. This research revealed that MMW irradiation could accelerate microtubule assembly, and this effect can be explained by temperature rise rather than by a non-thermal mechanism. In MMW irradiation of PC12 neuron-like cells, a slight increase in neurite growth and a slight increase in the accumulation of the dopamine metabolite 3,4-dihydroxyphenylacetic acid (DOPAC) were found, and the researchers suggest that this result is related to the thermal effect [63,64] (Figure 4a,b). The thermal effect caused by MMW irradiation and heat conduction has a different impact on the action potential emissivity of a single neuron and the characteristics of a single action potential [47]. 

The effect on the nervous system induced by MMW cannot be attributed entirely to thermal effects [93]. MMW irradiation could prevent or inhibit 75% of conditioned avoidance reflexes to animal pain [29]. This was due to changes in cell membrane surface charges, leading to activity changes of protein transport complex enzyme that affected microcirculation, coagulation, and the permeability of the vascular wall. It has also been proved that protein kinase was involved in the effects of MMW radiation on neutrophils [94]. Protein kinase is the key enzyme of the intracellular nerve information transmission process (protein phosphorylation). In the single-frequency MMW treatment study, MMW irradiation could achieve a better analgesic effect in skin areas with more nerve innervation and denser density [37,95]. This is because MMW radiation might activate the release of endogenous opioids [96]. In 2008, the same team tested this conclusion using direct ELISA measurements [35]. Then, they concluded that single-frequency MMW treatment had the best effect in inhibiting chronic non-neuropathic pain [97]. It is believed by both Sivachenko et al. [39] and Radzievsky et al. [96] that the peripheral and central nervous systems are jointly involved in the process of MMW-induced hypoalgesia. All in all, MMW will induce thermal or non-thermal effects on the epidermal jumpy system of organisms, accompanied by changes in action potentials or information substances, which will enable the central nervous system to receive signals and regulate neural activity, leading to the emergence of various distal effects.

### 7.2. Immune System

Earlier research by Logani et al. [30] on MMW radiation did not reverse cyclophosphamide (CPA)-induced immunosuppression. CPA significantly inhibited bone marrow and reduced the number of white blood cells (*p* < 0.05), but MMW radiation did not alleviate it. At the same time, they also confirmed that MMW therapy did not significantly reduce the catalase activity in mice’s blood [99]. However, their follow-up studies confirmed the effects of MMW irradiation on immune cells and the immune system. They believe that MMW irradiation affects T cells, macrophages, and NK cells differently. Makar et al. [25] studied the inhibition of T cell function by CPA and found that MMW irradiation could reverse this inhibition by increasing the activity of spleen cells and CD4+ cells. Among them, the levels of interleukin-10 (IL-10) and B cells were not affected by MMW irradiation, and the effect of MMW on immune function might be through the immune recovery process mediated by T cells [24]. Further research found that MMW can reverse the inhibitory effect of CPA on Th1 cytokines and enhance its expression [100]. This study initially confirmed that MMW may somewhat alleviate the damage of CPA on cellular immune function, but these conclusions still need more experimental verification and support. In their research into NK cells, Makar et al. [26] confirmed that NK cell activity can be stimulated by exposure to 42.2 GHz MMW irradiation. This study has once again confirmed that the regulation of MMW on the immune system may be based on the participation of endogenous opioids, and NK cells are the target of endogenous opioids. However, a research team believes that MMW may not necessarily positively impact the immune system. They believe that the low power density MMW at 100 GHz will increase the genomic instability of lymphocytes, which may increase the risk of cancer in ordinary people [101]. However, most of this research based on the above comes from the same research team, and the lack of repeatable research references will lead to the relative distortion of the research results.

MMW irradiation can increase the activity of neutrophils in blood [71]. Early studies have also shown that the power density of MMW affects the amount of ROS produced by neutrophils. Still, it is necessary to conduct more in-depth experimental and theoretical research on the structure and properties of the near-field region and eliminate the influence of thermal effect [71,98] (Figure 4c). However, follow-up studies have reached the opposite conclusion, and the ROS production induced by chemotactic peptide in resting cells was not changed by MMW irradiation [94] since protein kinase was the primary substance responsible for MMW-induced changes in neutrophil function. 

In short, for the immune system, MMW irradiation seems to reverse the inhibitory effect of CPA and enhance the activities of T cells [25,102], macrophages, and NK cells [26,103]. However, the results of existing studies on the effect of MMW irradiation on neutrophil activity are inconsistent. Further, in-depth and standardized research is needed to clarify its specific effect.

## 8. Research on MMW Biosafety

Nowadays, MMW is widely used in various fields, which causes people to “shuttle” in MMW irradiation every day. Therefore, it is of great significance to study the safety of MMW (Figure S1). A study has observed a correlation between high-frequency MMW irradiation and decreased muscle contraction intensity. However, further research is needed to confirm its causal relationship and potential mechanism [104]. MMW exposure may also affect the reproductive function of organisms and offspring. For example, it could induce delayed formation of immunodeficiency and even cause carcinogenic effects in offspring. Subbotina et al. [105] studied female and male rats, respectively, and found that MMW irradiation could cause harmful effects on pregnant rats and mutate the sperm of the experimental animal. In addition, the physiological solution treated with 4 Hz-modulated MMW could also lead to the contraction of isolated neurons and dehydration of brain tissue and skin [106]. A study has even suggested that high-frequency, low-power-density MMW irradiation may increase the risk of cancer in the general population [101].

Individual differences exist in the effects of MMW irradiation on cells; different cells will not have identical biological effects after MMW irradiation. MMW irradiation could enhance the growth rate of micro-colonies [106], reduce the nuclear charge, and increase chromatin agglutination [83]. Some studies have proved that MMW can produce reversible or irreversible damage to healthy tissues and cells.

Meanwhile, many studies have confirmed that MMW has no significant effect on healthy cells or tissue systems. Partial research confirmed that MMW has little impact at the cellular, subcellular, and molecular levels, and it could not change cell activity or induce gene expression [6,107,108]. For example, 1 mW/cm^2^ of 60 GHz MMW radiation does not affect the genetic toxicity in human cells [109]. Low-power MMW irradiation also did not affect the expression of stress and pain-related proteins and chaperonin [62,110]. Studies indicated that its irradiation also could not change the endoplasmic reticulum’s homeostasis and its protein’s folding and secretion [107,111]. Gene expression in human-related cells and cell viability were not affected by low-power MMW irradiation [112]. MMW alone also does not trigger endoplasmic reticulum stress in keratinocytes, the primary target cell [66]. The above studies were all performed at 60 Hz, and the MMW at 40 GHz was consistent with the data obtained at 60 Hz [113]. In studies on mouse peripheral blood and bone marrow cells, it was found that there was no evidence of a genotoxic effect of MMW radiation [114]. Additionally, 24 h of irradiation with 0.12 THz MMW at 5 mW/cm^2^ caused few or no effects on genotoxicity, morphology, and protein expression [115]. An in vitro study of human fibroblasts (HFFF2) showed that exposure to MMW did not cause genetic damage to HFFF2 cells [84]. Nearly the same conclusion has been reached in similar studies [42,116]. Low-power MMW exposure did not induce the production of heat shock protein 70 (Hsp70), and the cellular levels of RANTES and IP-10 were unchanged. Moreover, MMW irradiation did not seem to induce the apoptotic process of sperm [117]. 

Researchers are also trying to prove the safety of MMW not only at the cellular level but also at the organizational and systemic levels. For example, a low-power MMW at 60 GHz did not cause any detectable damage in rabbit eyes [118]. MMW did not inhibit mice’s gastrointestinal motility and transmission [96]. RF radiation at 94 GHz did not affect the incidence of tumors in the skin [65]. Despite the fact that a considerable number of subsequent studies yielded results that were contrary to the initial hypothesis, for instance, the application of MMW irradiation did not appear to reverse the inhibitory effect of CPA on the immune system [30]. However, the latest study has shown that low-power 101 GHz MMW irradiation does not harm healthy mice, and the irradiated mice’s physical, physiological, and case parameters are within the normal range [61]. In conclusion, this field still has great controversy and research space, although many studies have confirmed that MMW does not injure organisms.

## 9. Conclusions

Research on MMW biological effects is still facing tremendous difficulties and challenges. Looking back on the development track of MMW treatment in more than 70 years, there is research space but few related studies, many hot spots but few innovations, and a lot of controversies but little consensus. It is still in the stage of quantitative change accumulation before qualitative change, which seems to last for a long time. The main existing research results on MMW biological effects are as follows: (1) The primary mechanism of thermal effect is that high-power electromagnetic waves make water molecules move and collide to generate thermal energy. Although it is not regarded as the expected effect, its mechanism has been thoroughly studied. (2) The basic principle of low-frequency MMW treatment is that MMW radiation with corresponding frequency is applied to different resonance frequencies of various human body tissues, thereby causing resonance of the body tissues. (3) From the results, low-power MMW has specific treatment and inhibition effects on various inflammations, ulcers, tumor cells, and tumor tissues. It also plays a role in coordinating and repairing hematopoietic cells, chondrocytes, tissue regeneration, and immune function.

Several specific research groups completed the research on MMW biological effects, and the research directions of each research group are quite different. These scattered studies are not well correlated to provide a complete and systematic explanation of the non-thermal effects of MMW. In addition, there seems to be a wide range of controversy and discussion on the mechanism interpretation of the research results and even the results themselves. While most studies have demonstrated that MMW could inhibit the growth of “bad” cells and tissues and enhance the activity of “good” cells or systems, no studies have fully confirmed the safety of MMW. Finally, the lack of quality control in most studies makes the research results unconvincing.

Based on the diagnosis and treatment experience of biological effects by other physical factors, the ultimate breakthrough of the biological impact of MMW still depends on the in-depth exploration of cell signaling pathways. Researchers should explore the deep mechanism of MMW acting on cells and distancing from the existing signaling pathways. Meanwhile, prior experience should not be used, but full-spectrum studies should be used to determine the frequency selectivity of healthy or morbid cells for optimal frequency study coverage. Instead of focusing on pathological studies, more research is needed to determine healthy cells’ safety. Finally, researchers should improve the quality control of the study and increase the probability of obtaining favorable results and the repeatability of the experiment.

However, we recognize several limitations this review has that must be acknowledged. Firstly, despite our rigorous search strategy and selection process, the literature on MMWs is still relatively sparse and fragmented, with variable study quality, limiting our conclusions’ generalizability. Secondly, the complexity of MMWs’ mechanisms, particularly the non-thermal effects, and the interplay with cellular processes necessitates further exploration beyond the scope of this review. Thirdly, while we have emphasized the need for quality control, the lack of standardized protocols and measurement techniques in MMW research introduces an additional layer of uncertainty in interpreting the existing evidence. Lastly, the review primarily focuses on English-language literature, which might inadvertently exclude valuable contributions from non-English-speaking research communities.

## Figures and Tables

**Table 1:** MMW irradiation of tumor-associated cell studies, the wave source parameters, and their biological endpoints. Study of MMW irradiation of tumor cells (with positive results).

Tumor Cell or Tissue	Frequency	Power Density	Exposure Duration	Research Result	Research Group	Publication Year
RPMI 7932 human melanoma cells	52–78 GHz	-	1 h/s day, 3 h/day, up to 7 days	Growth inhibition	Beneduci et al. [13]	2005
MCF-7 human breast cancer cell	52–78 GHz	0.07 μW/cm^2^	-	Growth inhibition	Beneduci et al. [20]	2005
RPMI 7932 human melanoma cells	42.20, 53.57 GHz	0.3 mW/cm^2^	1 h/day, 4 days	No influence	Beneduci et al. [18]	2009
H1299 human lung cancer cells	75–105 GHz	0.2 mW/cm^2^	2, 4, or 10 min	Increase cell mortality and aging	Komoshvili et al. [27]	2020
H1299 human lung cancer cells and Non-tumorigenic MCF-10A epithelial cells	75–105 GHz	0.2 mW/cm^2^	2, 4, or 10 min	H1299 cells died, but MCF-10A cells had no effect	Komoshvili et al. [21]	2020
SW1353 human chondrosarcoma cells	33.2 ± 3 mm–45.6 ± 4 mm	4 mW/cm^2^	15, 30, 60, 90, and 120 min	Inhibit activity	Li et al. [22]	2012
Melanoma cells	58.4 GHz CW or PW	3.7 W	90 min pulse sequence (pulse duration 1.5 s and period 20 s)	Inducing cell damage	Orlacchio et al. [28]	2019
B16 F10 mouse melanoma cells	61.22 GHz	13.3 mW/cm^2^	15 min, 15 min/5 days	Growth inhibition	Radzievsky et al. [17]	2004
MCF-7 human breast cancer cell	50–75 GHz	Low-power	-	Growth inhibition	Samoilov et al. [29]	2015
A375 human melanoma cells	35.2 GHz	0.16 mW/cm^2^	90 min	Inhibit vitality and induce apoptosis	Zhao et al. [19]	2020

## Supplementary Materials

The following supporting information can be downloaded at: https://www.mdpi.com/article/10.3390/ijms25168638/s1.
