# Supplementary material for: Advances in Millimeter-Wave Treatment and Its Biological Effects Development
Source: Int J Mol Sci. 2024 Aug 8;25(16):8638. doi: 10.3390/ijms25168638 (PMC11354414; doi:10.3390/ijms25168638)
Supplement: Supplementary file 1 [file ijms-3115362-supplementary.pdf › ijms-3115362-supplementary.pdf]

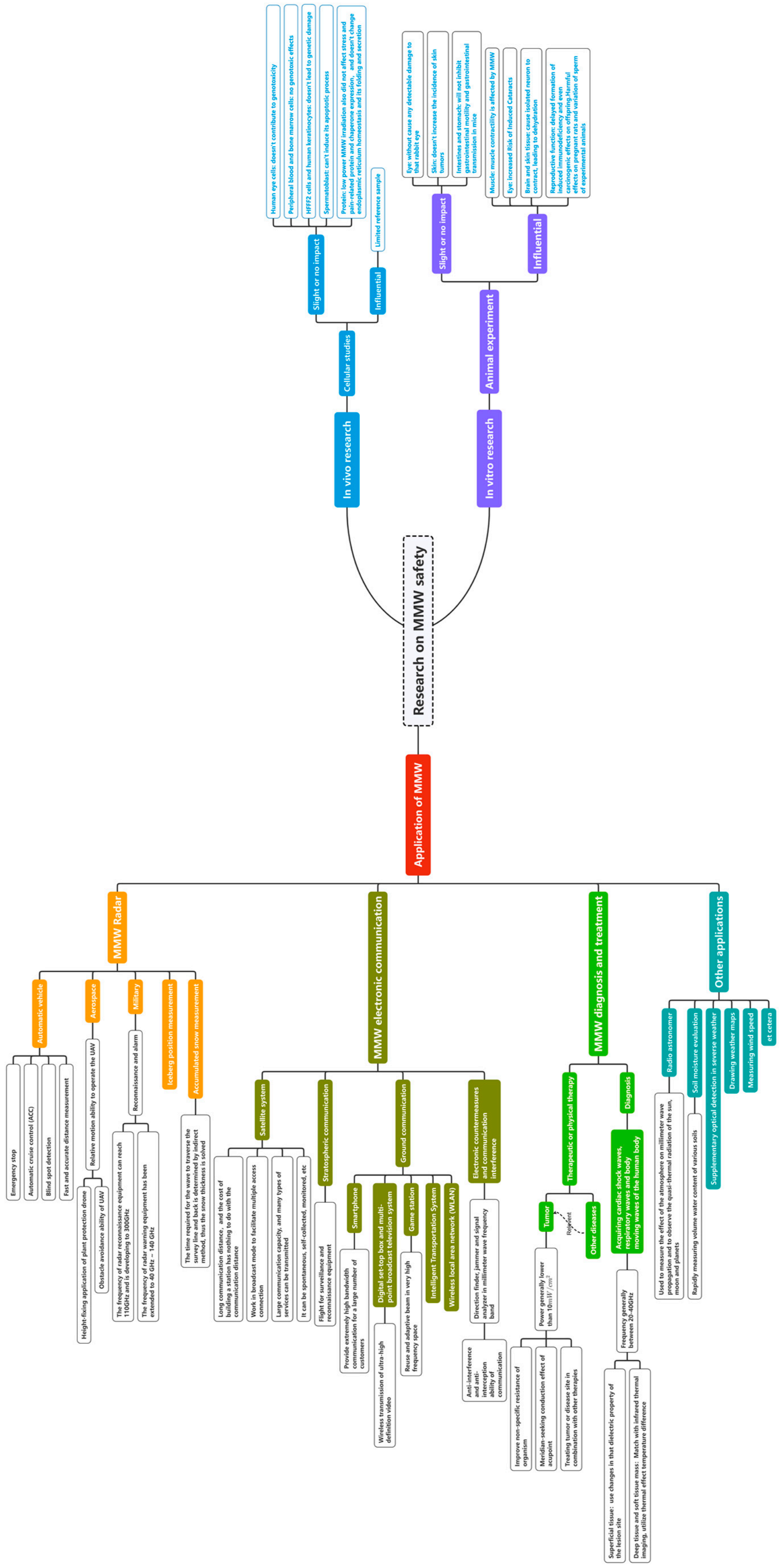

Figure S1. Comparison between MMW Applications and Safety Studies in Recent Years.
